# Genomic Evidence for Novel Introduction and Intra-Host Diversity of DENV-2 in Dar es Salaam, Tanzania

**DOI:** 10.3390/v18050585

**Published:** 2026-05-21

**Authors:** Silvan Hälg, Frank S. C. Tenywa, Nicole Liechti, Christian Beuret, Sarah J. Moore, Pie Müller

**Affiliations:** 1 Swiss Tropical and Public Health Institute, 4123 Allschwil, Switzerland; smoore@ihi.or.tz (S.J.M.); 2 Faculty of Science, University of Basel, 4051 Basel, Switzerland; 3 Ifakara Health Institute, Bagamoyo P.O. Box 74, Tanzania; 4 Spiez Laboratory, 3700 Spiez, Switzerland; nicole.liechti@babs.admin.ch (N.L.); 5 Nelson Mandela African Institute of Science and Technologys, P O BOX 447 Tengeru, Tanzania

**Keywords:** dengue virus, *Aedes aegypti*, arbovirus surveillance, nanopore sequencing, phylogenetic analysis, viral evolution, genomic epidemiology

## Abstract

Dengue virus (DENV) poses a growing risk in Tanzania, yet its genetic diversity in mosquito populations remains poorly understood. Using Nanopore sequencing, we recovered full coding sequences from six DENV-2 positive mosquito pools collected in Dar es Salaam outside recognized outbreak periods. Phylogenetic analysis placed these sequences in a distinct monophyletic clade within genotype II, separate from strains linked to Tanzania’s 2014 outbreak. Instead, they clustered with Asian lineages and showed the closest relatedness to DENV-2 strains from Kenya (2013) and India (2014), with divergence estimated to have occurred around 2010. Variant profiling identified 212 low-frequency intra-pool variants, predominantly non-synonymous changes in the NS3, NS4B, and NS5 coding regions. These results suggest a previously unrecognized introduction of genotype II that is now circulating silently within local mosquito populations. Our findings highlight the value of genomic surveillance in mosquito vectors for early detection of arboviral threats, even in the absence of reported human cases.

## 1. Introduction

Dengue virus (DENV) is a mosquito-borne virus belonging to the species *Orthoflavivirus denguei*, genus *Orthoflavivirus*, family Flaviviridae. It is associated with a wide spectrum of clinical manifestations ranging, from mild fever to severe disease forms such as dengue hemorrhagic fever and dengue shock syndrome [1]. Worldwide, DENV is estimated to infect approximately 390 million people each year, with nearly 96 million individuals developing clinical symptoms, making it one of the most widespread mosquito-borne viral infections globally [2,3]. The rapid global expansion of dengue has been fueled by urbanization, climate change, and increasing international travel and trade [2,4,5].

Four antigenically distinct serotypes (DENV-1 to DENV-4) circulate globally, including in endemic urban settings such as Dar es Salaam, and each is capable of causing the full clinical disease spectrum [6]. While primary infection provides long-term immunity against the specific serotype, heterologous secondary infections can result in more severe disease through antibody-dependent enhancement [7,8]. Each serotype is further subdivided into multiple genotypes with distinct geographical distributions and epidemiological patterns. Although updated lineage nomenclature systems have been proposed to improve the resolution of genomic surveillance, classical genotype classification remains widely used in molecular epidemiological studies [9].

Molecular epidemiology, particularly whole-genome sequencing, has become essential for tracing DENV introduction, lineage replacement, and viral adaptation to both vectors and hosts [10,11]. Phylogenetic studies increasingly document intercontinental virus movement, with growing evidence of Asian DENV genotypes being introduced into Africa [12,13].

In Tanzania, dengue has emerged as a recurrent public health concern over the past decade, with outbreaks reported primarily in coastal urban centers, particularly Dar es Salaam [13,14]. The city’s high population density, rapid urban expansion, and favorable climatic conditions support sustained transmission of *Aedes aegypti*, the principal dengue vector [15]. Since the first confirmed outbreak in 2010, Dar es Salaam has experienced repeated dengue epidemics of varying magnitude, with evidence suggesting periodic serotype replacement and introduction of new viral lineages [13]. In addition to epidemic waves, accumulating evidence indicates the presence of inter-epidemic transmission, suggesting that dengue virus may persist at low levels between recognized outbreaks [15].

Despite this, Africa, and Tanzania in particular, remains underrepresented in genomic surveillance datasets. Tanzania has witnessed multiple dengue outbreaks since 2010, including major episodes in 2014 (predominantly DENV-2 genotype II, Cosmopolitan) and 2019 (DENV-1) [12]. These outbreaks have been phylogenetically linked to Asian countries such as China and Singapore [12]. However, few studies have sequenced viruses directly from mosquito vectors, and information on viral evolution within mosquito reservoirs remains limited.

In parallel, growing evidence suggests that intra-host viral diversity, especially within non-structural genes (NS) like NS3 and NS5, plays a vital role in dengue adaptive evolution. Studies in both human and mosquito hosts have identified selection pressures acting on NS genes during natural DENV infection [16]. Such intra-host diversity has been linked to viral replication fitness and quasi-species dynamics, emphasizing its relevance for surveillance and prediction of transmission risk [17].

In a previous longitudinal xenomonitoring study, DENV-2 has been detected in *Ae. aegypti* mosquitoes across Dar es Salaam during inter-epidemic period, indicating silent, persistent urban transmission [15]. Such low-level transmission in the absence of clinical cases is increasingly recognized as a crucial factor in maintaining viral circulation and potentially seeding future epidemics [14,18]. To better understand the evolutionary origins, genomic diversity, and potential introduction routes of these mosquito-derived DENV-2 strains, we applied Oxford Nanopore sequencing to generate whole genomes from six DENV-2–positive mosquito pools.

To our knowledge, this study provides the first detailed genomic and phylogenetic characterization of DENV-2 circulating within mosquito vectors in Dar es Salaam, Tanzania. By integrating whole-genome and gene-specific phylogenies with intra- and inter-sample variant analyses, we aimed to determine whether these viruses represent ongoing local lineages or newly introduced genotypes. Our findings provide critical data for enhancing genomic surveillance strategies and inform public health authorities in Tanzania and the wider East African region.

## 2. Materials and Methods

### 2.1. Sample Origin and RNA Extraction

A total of six DENV-2–positive mosquito pools were selected for sequencing. Five viruse-positive pools were identified by reverse transcription quantitative PCR (RT-qPCR) during a previously described two-year longitudinal surveillance study conducted in Dar es Salaam, Tanzania [15]. Mosquitoes were collected between 2023 and 2024 and processed in pools of 10 individuals. Dar es Salaam is a densely populated coastal urban center that has experienced recurrent dengue virus activity since 2010, with evidence of both outbreak-associated and inter-epidemic transmission. Detailed mosquito sampling procedures and site-level collection information have been previously described [15].

The sixth DENV-2–positive sample was detected in a mosquito pool collected in 2022 from the same study area but processed independently. For this sample, 600 µL of InhibitEx Buffer (Qiagen AG, Hilden, Germany) was added to a pool of 10 mosquitoes. The sample was homogenized twice for 30 s using a QIAGEN TissueLyser II (Qiagen AG, Hilden, Germany), then centrifuged at 3220× *g* for 5 min. A 100 µL aliquot of the supernatant was mixed with 400 µL AVL buffer (Qiagen AG, Hilden, Germany). Nucleic acid extraction was performed using the DNA and Viral NA Large Volume Kit (Roche Diagnostics, Rotkreuz, Switzerland) on a MagNa Pure 96 System (Roche Diagnostics, Rotkreuz, Switzerland). The remaining five DENV-2-positive samples were processed as described previously [15].

### 2.2. cDNA Synthesis and Amplicon Generation

Extracted RNA was reverse-transcribed using SuperScript IV Reverse Transcriptase (Thermo Fisher Scientific, Waltham, MA, USA) with random hexamers. Whole genome amplification of DENV-2 was performed using a tiling multiplex PCR approach. Primer sequences were used as published by Su et al. [19], enabling serotype-specific multiplex amplification of dengue virus genomes directly from clinical or entomological samples. The primers generated ~400 bp overlapping amplicons covering the entire coding region.

### 2.3. Library Preparation and Nanopore Sequencing

PCR products were purified using AMPure XP beads (Bioconcept AG, Allschwil, Switzerland). Purified amplicons were barcoded using the Native Barcoding Expansion Kit and prepared for sequencing with the Ligation Sequencing Kit (SQK-LSK114) (Oxford Nanopore Technologies, Oxford, UK). Libraries were loaded onto a MinION R10.4.1 flow cell (Oxford Nanopore Technologies, Oxford, UK) and sequenced on a GridION platform (Oxford Nanopore Technologies, Oxford, UK) using MinKNOW software v5.8.12 for live base-calling (Guppy v6.4.2, high-accuracy mode), real-time demultiplexing, and quality filtering (Q > 10).

### 2.4. Genome Assembly and Consensus Generation

Primer sequences were trimmed from raw reads using cutadapt v4.9 [20]. Reads were quality-filtered (>Q20) with Filtlong v0.3.0 [21] and mapped to a reference DENV-2 reference genome (GenBank: MT982148.1) using Minimap2 v2.28-r1209 [22], followed by indexing with SAMtools v1.22.1 [23]. Consensus sequence generation and variant calling were performed using iVar v1.4.3 [24]. Positions with coverage below 10× were masked with “N” to ensure high-quality consensus sequences. Genome completeness was assessed by calculating the percentage of ambiguous nucleotides (Ns) per sequence (Table 1). Complete genome sequences were submitted to GenBank under accession numbers PV834971–PV834976.

### 2.5. Multiple Sequence Alignment and Phylogenetic Analysis

To determine DENV-2 genotype identity and explore evolutionary relationships, three phylogenetic analyses were performed: (i) genotype assignment using representative reference genomes from the six recognized DENV-2 genotypes (I–VI), (ii) global phylogenetic contextualization of genotype II complete genomes, and (iii) envelope (*E*) gene–based phylogenetic resolution within circulating lineages.

Sequences were aligned using MAFFT v7 [25]. Phylogenetic trees were reconstructed using BEAST v2.7.7 [26] under a generalized time reversible model with gamma-distributed rate variation (GTR+Γ) across four categories. The GTR+Γ model was selected because it provides a flexible framework for modeling nucleotide substitution patterns commonly observed in RNA virus genomes.

A relaxed log-normal molecular clock model was applied to accommodate lineage-specific rate heterogeneity, with an initial mean clock rate of 0.001 substitutions/site/year based on published dengue virus estimates [27]. To avoid imposing a fixed demographic structure, a Bayesian Skyline coalescent prior was used, allowing the effective population size to vary over time.

XML input files were generated using BEAUti from the BEAST software package. Markov Chain Monte Carlo (MCMC) chains were run for 100 million generations. Convergence and effective sample size (ESS) values were assessed using Tracer v1.7.2 [28], with ESS > 200 considered adequate. After discarding 10% burn-in, maximum clade credibility (MCC) trees were generated using TreeAnnotator within the BEAST package and visualized using FigTree v1.4.4 [29].

For genotype determination, curated reference genomes representing all six DENV-2 genotypes were included. For genotype II contextualization, 336 unique complete genomes were retrieved from GenBank after removing duplicate and low-quality sequences. For *E* gene phylogenetics, 1132 unique genotype II *E* gene sequences were compiled from GenBank, complete genome datasets, and the six sequences generated in this study.

Temporal phylogenetic analyses incorporated sampling dates when available. MCC trees were rooted using a historical DENV-2 genotype II reference genome (GenBank accession KM204118.1).

The *E* gene was analyzed separately because its higher sequence variability and immune-driven selection improve phylogenetic discrimination. The relatively large number of publicly available *E* gene sequences also facilitates robust comparative evolutionary analysis. Additional phylogenetic trees and methodological information are provided in the Supplementary Materials.

### 2.6. Variant Analysis

To characterize within-sample viral diversity, reads from each mosquito pool were aligned to a sample-specific consensus genome generated from the same dataset to reduce mapping bias and improve sensitivity for low-frequency variants. Primer sequences were trimmed using cutadapt v4.9 [20] and reads were aligned using Minimap2 v2.24 [22]. Alignment output processing, sorting and indexing were conducted with SAMtools v1.14 [23]. Variants were called using the iVar pipeline v1.4.3 [24] with a minimum base quality threshold of 20, retaining only variants marked with the “PASS” flag.

Additional filtering criteria included minimum read depth ≥400× and alternate allele frequency ≥5% to reduce stochastic noise and nanopore-specific sequencing artifacts. Only canonical nucleotide substitutions (A, T, G, or C) were retained, while ambiguous calls, indels, and stop codons were excluded.

Variants were classified as synonymous or nonsynonymous by comparing reference and alternate amino acids. Codon positions were translated into global polyprotein coordinates based on gene annotations. Processed variant data were analyzed and visualized in R v4.5.1 using ggplot2 [30], tidyverse [31], and ComplexUpset [32,33]. A custom schematic of the dengue virus polyprotein, depicting structural (capsid, precursor membrane, envelope) and nonstructural (NS1–NS5) proteins, was overlaid beneath variant distribution plots to provide functional context.

## 3. Results

### 3.1. Nanopore Sequencing

Nanopore sequencing enabled recovery of full-length dengue virus genomes (>10,000 nt) from all six DENV-2–positive mosquito pool samples. Sequencing performance metrics, including raw read counts, filtered reads, mapping rates, and coverage statistics, are summarized in Table 1. Across samples, raw sequencing output ranged from approximately 260,000 to 5,000,000 reads per sample, and the high genome coverage depth supported robust downstream phylogenetic and variant analyses.

### 3.2. Phylogenetic Analysis

**Figure 1:**
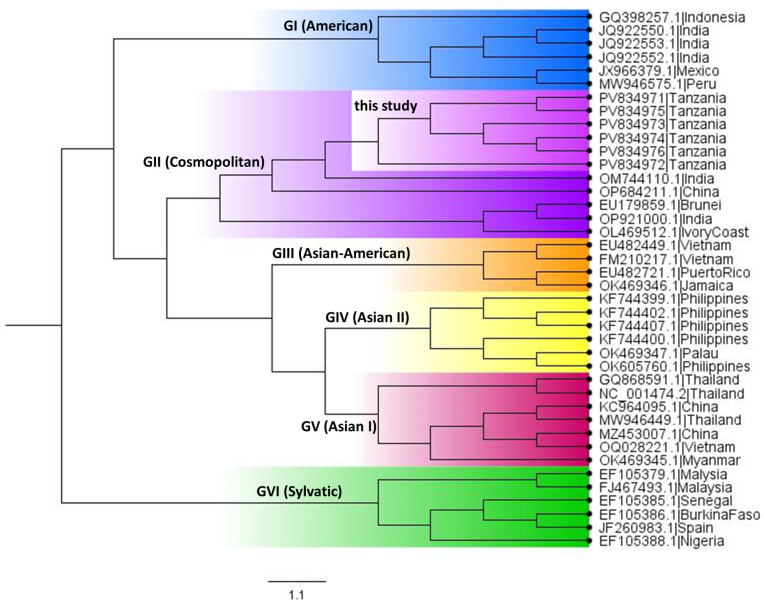
Phylogenetic tree of DENV-2 genotypes inferred from complete genome sequences. The tree was reconstructed from consensus complete genome sequences generated in this study together with representative reference genomes spanning all recognized DENV-2 genotypes. Phylogenetic inference was performed in BEAST under a generalized time-reversible (GTR) nucleotide substitution model with gamma-distributed rate variation across four categories (GTR+G4). A relaxed log-normal molecular clock with a mean clock rate of 0.001 substitutions/site/year was applied and a Bayesian Skyline coalescent prior were applied. Markov Chain Monte Carlo (MCMC) sampling was run for 100 million iterations to ensure convergence and adequate posterior sampling.

To determine the genotypes of the newly sequenced DENV-2 samples from Dar es Salaam, a phylogenetic tree was constructed using a reference panel representing the known diversity of DENV-2 genotypes. All six samples clustered within the genotype II clade, confirming their genotype assignment (Figure 1). This initial classification provided a robust framework for subsequent evolutionary and epidemiological analyses.

The analysis was expanded by incorporating 336 globally sampled, unique, complete DENV-2 genotype II genome sequences. Here, “unique” refers to the removal of duplicate or highly redundant sequences from public databases, whereas “complete genomes” were defined as sequences covering the full coding region with minimal ambiguous nucleotide content. Using tip-dated sequences and a relaxed molecular clock model, a time-scaled phylogeny was inferred to resolve the temporal evolutionary relationships within genotype II. The estimated mean substitution rate was 9.17 × 10^−4^ substitutions/site/year (95% highest posterior density [HPD]: 8.24 × 10^−4^–1.01 × 10^−3^).

**Figure 2:**
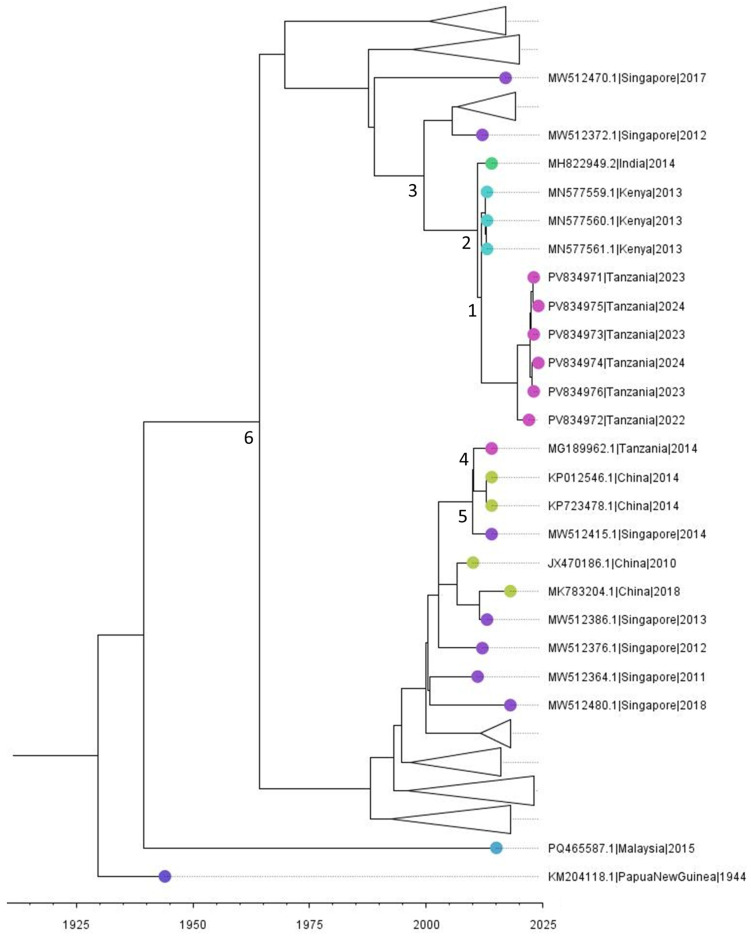
Time-scaled phylogenetic tree of DENV-2 genotype II complete genome sequences. The tree was inferred under a generalized time-reversible (GTR) nucleotide substitution model with gamma-distributed rate variation across sites discretized into four categories (GTR+G4). A relaxed uncorrelated log-normal molecular clock model was applied, with the mean clock rate fixed at 0.001 substitutions/site/year. Demographic changes over time were modelled using a Bayesian Skyline coalescent prior. The Markov Chain Monte Carlo (MCMC) sampling was run for 100 million iterations, with sampling every 10,000 steps and the first 10% discarded as burn-in. Convergence was confirmed by effective sample size (ESS) > 200 for all parameters. Posterior probabilities and 95% highest posterior density (HPD) intervals support divergence time estimates. Tips are colored by country of origin to illustrate the geographic distribution of sequences. 1: 2011 (95% HPD: 2011–2012); 2: 2010 (95% HPD: 2009–2011); 3: 1999 (95% HPD: 1994–2003); 4: 2010 (95% HPD: 2009–2012); 5: 2009 (95% HPD: 2009–2012); 6: 1964 (95% HPD: 1954–1976).

All Tanzanian DENV-2 sequences generated in this study clustered on a single terminal branch but remained distinct from previously reported Tanzanian complete genomes collected in 2014. Phylogenetic reconstruction suggests that this lineage began diverging as early as 1964 (95% HPD: 1954–1976) (Figure 2). Instead, the newly characterized isolates demonstrate closer phylogenetic relatedness to strains from Kenya (2013) and India (2014). Molecular clock analyses dates divergence between the new Tanzanian and Kenyan lineages to approximately 2011 (95% HPD: 2011–2012), with both sharing a common ancestor with the Indian isolate around 2010 (95% HPD: 2009–2011). A more ancestral node connects these sequences to a Singaporean isolate, with an estimated divergence around 1999 (95% HPD: 1994–2003) (Figure 2).

To further evaluate the phylogenetic signal within a functionally important genomic region, a parallel phylogenetic analysis using *E* gene sequences was conducted. The *E* gene was selected because of its widespread use in dengue virus molecular epidemiology, driven by its relatively high sequence variability and the broad availability of reference sequences in public databases. The resulting topological and divergency patterns are consistent with the complete genome phylogeny, providing independent support for the evolutionary relationships inferred in this study (Supplementary Figure S1).

### 3.3. Variant Analysis

**Figure 3:**
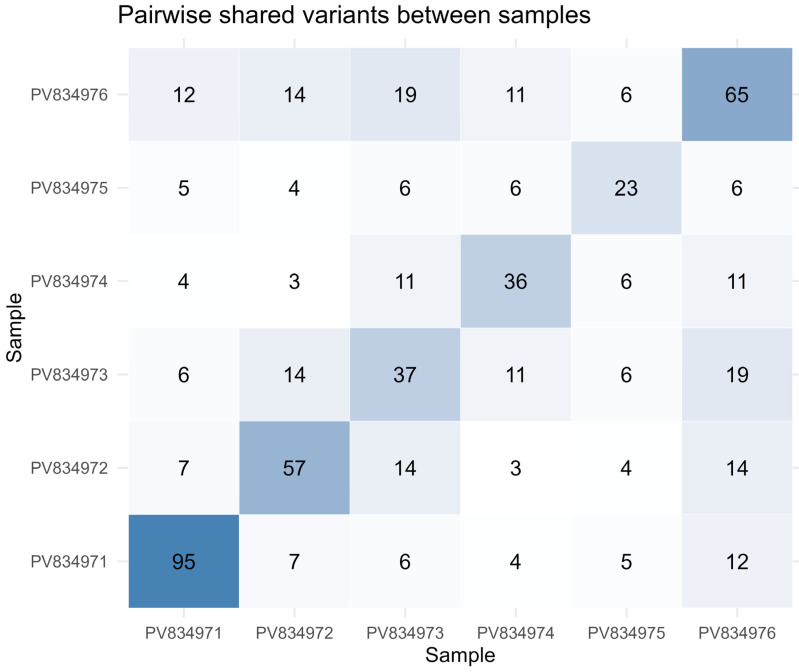
Pairwise shared nucleotide variants among DENV-2–positive mosquito pool samples. The heatmap shows the number of shared nucleotide variants between each pair of samples, based on unique genomic positions and base substitutions. Diagonal values represent the total number of variants detected per sample, while off-diagonal values indicate the number of variants shared between sample pairs. Color intensity reflects the magnitude of shared variation.

Using a consensus alignment strategy, in which sequencing reads were realigned to their corresponding sample-specific consensus genomes, a total of 212 within-pool variants across the six DENV-2–positive mosquito samples were identified. Of these, 170 variants were private to individual pools, whereas the remaining variants were shared by two or more samples (Figure 3, Supplementary Figure S2). Mutations detected in three or more samples are summarized in Table 2, and a complete list of all variants, including sample-specific mutations, is provided in Supplementary Table S1.

**Figure 4:**
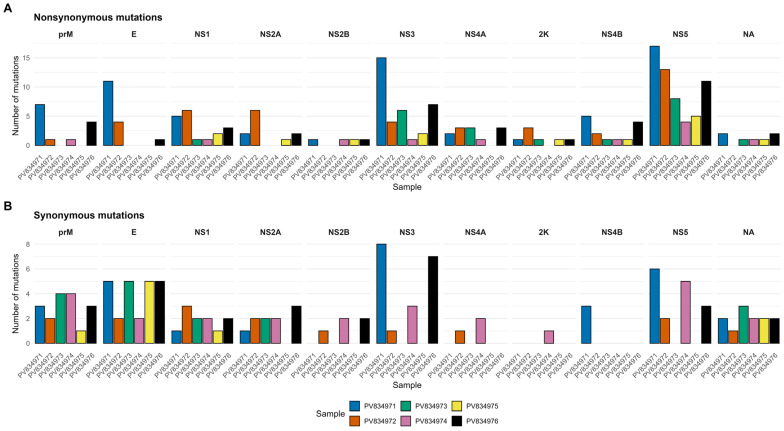
Distribution of nonsynonymous (**A**) and synonymous (**B**) mutations across DENV-2 genomic regions in the six sequenced mosquito pool samples. Mutation counts are shown for each gene using a unified y-axis scale to facilitate comparison across samples. prM: Premembrane protein, E: Envelope protein, NS1: Non-structural protein 1, NS2A: Non-structural protein 2A, NS2B: Non-structural protein 2B, NS3: Non-structural protein 3, NS4A: Non-structural protein 4A, 2K: 2K peptide, NS4B: Non-structural protein 4B, NS5: Non-structural protein 5, NA: Not available.

Within individual samples, most variants occurred at allele frequencies between 5% and 20%, consistent with low-frequency variants that may represent minor viral subpopulations within pooled mosquitoes (Supplementary Figures S3–S8). Nonsynonymous mutations were broadly distributed across the viral polyprotein and outnumbered synonymous mutations in most samples. These mutations were particularly frequent in nonstructural genes, including NS3, NS4B, and NS5 (Figure 4 and Supplementary Figures S3–S8), suggesting elevated codon diversity within these genomic regions at the within-pool level.

## 4. Discussion

This study reports the detection of DENV-2 genotype II in *Ae. aegypti* mosquitoes collected between outbreaks in Dar es Salaam, Tanzania. Further analysis revealed a complex landscape of low-frequency genetic diversity within the mosquito-derived DENV-2 populations, with several predominating nonsynonymous mutations in the NS3, NS4B, and NS5 coding regions.

Comparative genomic and phylogenetic analyses revealed that the newly identified Tanzanian DENV-2 genotype II isolates form a distinct cluster separate from strains associated with the 2014 outbreak [34]. Instead, the new isolates cluster with Keny (2023) and India (2014), supporting the hypothesis of multiple temporally staggered introductions into the region [35]. Molecular clock analyses indicate that the Tanzanian sequences reported here share a most recent common ancestor with Asian lineage viruses around 2009–2011. This divergence predates the lineage responsible for the 2014 outbreak in Tanzania, which instead clusters with viruses from China and Singapore sampled in 2014. Our sequences therefore represent an independent introduction distinct from the 2014 outbreak lineage [13]. The most recent common ancestor of the two lineages found in Tanzania dates to around 1964. The deep divergence further supports the hypothesis of independent introductions with distinct epidemiological histories, consistent with broader regional arbovirus surveillance efforts [2].

A similar pattern of viral flow between East Africa and South Asia has been documented previously [12], and recent studies from Kenya and Djibouti have documented diverse DENV-2 lineages, some also closely related to Asian strains [12,36,37,38]. Altogether, these findings emphasize that East Africa serves as a convergence point for interregional dengue virus traffic, with complex and dynamic transmission patterns across the region [39]. The observation of at least two independent introductions of DENV-2 genotype II into Tanzania parallels patterns seen in other hyperendemic regions, such as Brazil and Southeast Asia, where multiple co-circulating lineages within a single serotype are common [40,41]. These data support the hypothesis that long-range viral movement, likely facilitated by international travel and trade, is a major driver of dengue virus genetic diversity in Africa.

The predominance of nonsynonymous over synonymous variants suggests selection for specific traits, such as immune evasion or adaptations to tissue-specific environments within the mosquito vector [16,42]. In the present study, nonsynonymous variants were particularly enriched in genomic sequences encoding for the nonstructural proteins NS3, NS4B, and NS5. This pattern aligns with previous evidence identifying these regions as hotspots for functional adaptations in flaviviruses [16,42,43]. Notably, NS5, which encodes the RNA-dependent RNA polymerase, is known to undergo adaptive changes that influence replication fidelity and immune evasion [44]. The observed within-pool evolutionary pattern aligns with broader studies on RNA virus evolution showing that elevated intrahost diversity can promote the emergence of fitter variants that eventually contribute to outbreaks [16,42]. Therefore, viral evolution within vectors represents a critical, yet often underappreciated, component of arboviral epidemiology [44,45,46,47].

The present findings emphasize the importance of continuous molecular surveillance to detect new viral introductions before they trigger outbreaks. The genetic divergence between currently circulating isolates and those in 2014 may also have implications for diagnostic accuracy and local immunity, particularly given the elevated risk of severe disease associated with secondary DENV-2 infections [48,49].

A key limitation of this study is that viral sequencing was performed on pools of 10 mosquitoes rather than on individual specimens. As a result, the observed within-sample diversity may reflect not only true intra-host viral diversity within individual mosquitoes but also mixed infections among different mosquitoes within the same pool. If multiple mosquitoes within a pool carried genetically distinct DENV-2 variants, estimates of apparent intra-host diversity may be inflated. Accordingly, the variation reported here should be interpreted as within-pool diversity rather than strictly intra-host diversity. Future studies sequencing individual mosquitoes will be important for disentangling true within-host viral evolution from inter-host variation introduced by pooled sampling.

Several additional limitations should be acknowledged. Although screening was performed for all four DENV serotypes throughout the study period, only six DENV-2 genotype II samples were detected and sequenced. This limited number of samples restricts the diversity captured and may not fully represent the broader circulating viral population. The absence of contemporaneous clinical genomes further limits the ability to directly connect vector-derived viral diversity with human infections and transmission dynamics. Temporal sampling gaps also restrict resolution of local evolutionary trajectories.

Future research should prioritize larger sample sizes, sequencing of individual mosquitoes and human cases, and integration of epidemiological metadata to better resolve transmission networks and viral evolution. Longitudinal studies that combine vector and human surveillance will be essential for identifying drivers of viral persistence and emergence. Additionally, functional characterization of nonsynonymous mutations, particularly in NS3, NS4B, and NS5, will be critical for understanding their effects on viral fitness, vector competence, and immune evasion.

## 5. Conclusions

In conclusion, this study highlights the value of entomological genomic surveillance for early detection of emerging arboviral threats, even in the absence of reported human cases. Effective dengue control will require integration of human case monitoring with vector-based genomic surveillance. Such integrated approaches are essential for revealing mechanisms of viral persistence, adaptation, and spread, thereby enabling more targeted interventions against both the virus and its mosquito vectors. Our findings further emphasize the need for sustained genomic surveillance in Tanzania, where the transmission landscape remains complex and dynamic, to better anticipate and mitigate future outbreaks.

## Figures and Tables

**Table 1:** Summary of sequencing metrics for each sample.

Sample ID	Raw Reads	Filtered Reads	Mapping Rate (%)	Mean Depth of Coverage	Genome Coverage (%)	% Ns
PV834971	265,582	262,170	80.0	549.6	100.0	0.87
PV834972	880,081	876,263	86.5	25,428.8	100.0	0.10
PV834973	4,784,591	4,736,336	88.2	144,889.0	100.0	0.009
PV834974	4,302,475	4,293,672	93.1	127,332.0	100.0	2.11
PV834975	4,950,109	4,945,016	88.0	140,270.0	98.5	4.08
PV834976	469,672	463,328	78.0	12,229.9	100.0	0.07

**Table 2:** Shared mutations detected in three or more DENV-2–positive mosquito pool samples.

Sample ID	Pos nt	Ref nt	Alt nt	Alt freq	Total Depth	*p*-Value	GFF Feature	Ref aa	Alt aa	Pos aa
PV834971	397	C	T	0.233763	11,409	0	ancC:cds-ancC	I	I	109
PV834972				0.0722392	309,740	0				
PV834973				0.308155	27,492	0				
PV834974				0.246182	261,794	0				
PV834975				0.32994	409,993	0				
PV834976				0.258511	20,827	0				
PV834971	385	C	T	0.169001	11,639	0	ancC:cds-ancC	I	I	105
PV834973				0.313835	355,400	0				
PV834974				0.259055	320,245	0				
PV834975				0.290453	463,129	0				
PV834976				0.205332	22,768	0				
PV834971	382	G	A	0.351974	7725	0	ancC:cds-ancC	M	I	104
PV834973				0.325035	345,224	0				
PV834974				0.265117	313,994	0				
PV834975				0.29478	457,717	0				
PV834976				0.345582	17,472	0				
PV834971	808	T	C	0.285965	6826	0	prM:cds-prM	G	G	132
PV834973				0.389211	268,654	0				
PV834974				0.267773	295,881	0				
PV834976				0.30256	12,576	0				
PV834973	1759	C	T	0.143156	514,321	0	E:cds-E	L	L	283
PV834974				0.101009	633,361	0				
PV834975				0.16945	296,606	0				
PV834976				0.103475	19,048	0				
PV834971	3953	C	T	0.474026	154	3.43 x 10^-38^	NS2A:cds-NS2A	L	L	168
PV834972				0.33045	578	2.15 x 10^-173^				
PV834973				0.106201	51,412	0				
PV834971	697	C	T	0.131119	1144	1.94 x 10^-112^	prM:cds-prM	L	L	95
PV834972				0.0615836	341	9.22 x 10^-11^				
PV834976				0.0512948	4016	4.54 x 10^-68^				
PV834972	9350	A	G	0.149415	4270	0	NS5:cds-NS5	S	G	603
PV834973				0.425261	92,703	0				
PV834976				0.387881	3251	0				
PV834972	8340	G	A	0.161336	1407	1.04 x 10^-145^	NS5:cds-NS5	S	N	266
PV834973				0.183547	42,120	0				
PV834976				0.171831	3195	2.00 x 10^-223^				
PV834973	2810	G	A	0.0816028	119,812	0	NS1:cds-NS1	E	K	139
PV834974				0.0909034	47,633	0				
PV834976				0.207317	2542	2.70 x 10^-287^				
PV834973	2680	A	T	0.452809	85,462	0	NS1:cds-NS1	G	G	95
PV834974				0.392176	32,006	0				
PV834976				0.299807	3102	0				
PV834973	2674	C	T	0.341153	77,118	0	NS1:cds-NS1	I	I	93
PV834974				0.0794454	29,064	0				
PV834976				0.313271	2675	0				

The table summarizes nucleotide substitutions identified in ≥3 of the six sequenced pools after quality filtering. Reported metrics include nucleotide position (Pos), reference (Ref nt) and alternate (Alt nt) alleles, the number of samples sharing each mutation (n/6), allele frequency (Alt freq), sequencing depth, statistical support (*p*-value), genomic annotation (GFF feature), and the corresponding amino acid changes (Ref aa and Alt aa). A complete list of all detected mutations, including sample-specific variants, is provided in the Supplementary Materials.

## Data Availability

The collection and use of mosquito specimens in this study were conducted in compliance with the Nagoya Protocol on Access and Benefit-Sharing. Sampling and export of mosquito material were authorized by the Tanzanian authorities under research permit No. #IHI/CED/DSM/2022/476, issued by the Vice President’s Office. The subsequent transport of specimens to Switzerland for viral metagenomic analysis was carried out in accordance with the terms of this permit and applicable international regulations. Complete genomes from the six dengue viruses are available on GenBank under accession numbers PV834971, PV834972, PV834973, PV834974, PV834975 and PV834976.

## Supplementary Materials

The following supporting information can be downloaded at https://www.mdpi.com/article/10.3390/v18050585/s1: Supplementary File, Supplementary Table.

## Abbreviations

The following abbreviations are used in this manuscript:

*C*	Capsid gene
DENV	Dengue virus
*E*	Envelope gene
GTR	Generalized Time Reversible
HPD	Highest Posterior Density
MCC	Maximum Clade Credibility
MCMC	Markov Chain Monte Carlo
*NS*	Non-structural gene
ONT	Oxford Nanopore Technology
*prM*	Precursor membrane protein gene
