# Supplementary material for: Genomic Evidence for Novel Introduction and Intra-Host Diversity of DENV-2 in Dar es Salaam, Tanzania
Source: Viruses. 2026 May 21;18(5):585. doi: 10.3390/v18050585 (PMC13211437; doi:10.3390/v18050585)
Supplement: Supplementary file 1 [file viruses-18-00585-s001.zip › viruses-4171315-supplementary.pdf]

viruses

MDPI

Supplementary File

Genomic Evidence for a Novel Introduction and Intra-Host Diversity of DENV-2 in Dar es Salaam, Tanzania

Silvan Hälg [1,2], Frank S.C. Tenywa [1,2,3], Nicole Liechti [4], Christian Beuret [4], Sarah J. Moore [1,2,3,5] and Pie Müller [1,2]

1 Swiss Tropical and Public Health Institute, 4123 Allschwil, Switzerland; smoore@ihi.or.tz (S.J.M.)
2 University of Basel Faculty of Science, 4051 Basel, Switzerland
3 Ifakara Health Institute, Bagamoyo P.O. Box 74, Tanzania
4 Spiez Laboratory, 3700 Spiez, Switzerland; nicole.liechti@babs.admin.ch (N.L.)
5 Nelson Mandela African Institute of Science and Technologys, Tengeru P.O. Box 447, Tanzania
* Correspondence: silvan.haelg@swisstph.ch (S.H.); pie.mueller@swisstph.ch (P.M.)

Materials and Methods

E Sequence Alignment and Phylogenetic Analysis

To increase phylogenetic resolution within the E gene, 782 unique E protein sequences from the DENV-2 genotype II were retrieved from GenBank, and E gene regions were extracted from the 336 full genome sequences and from our six newly generated sequences. After removing duplicates, 1,132 unique E gene sequences were included in the alignment using MAFFT v7 (1). Phylogenetic inference followed the same pipeline as for the complete genome approach, except the HKY substitution model was used with gamma rate heterogeneity (four categories), and a 20% burn-in was applied when generating the MCC tree. As with the full genome analysis, the E gene tree was rooted to the 1944 DENV-2 genotype II reference (KM204118.1) to enable consistent evolutionary comparison. Final tree visualizations were produced using FigTree v1.4.4 (2).

Results

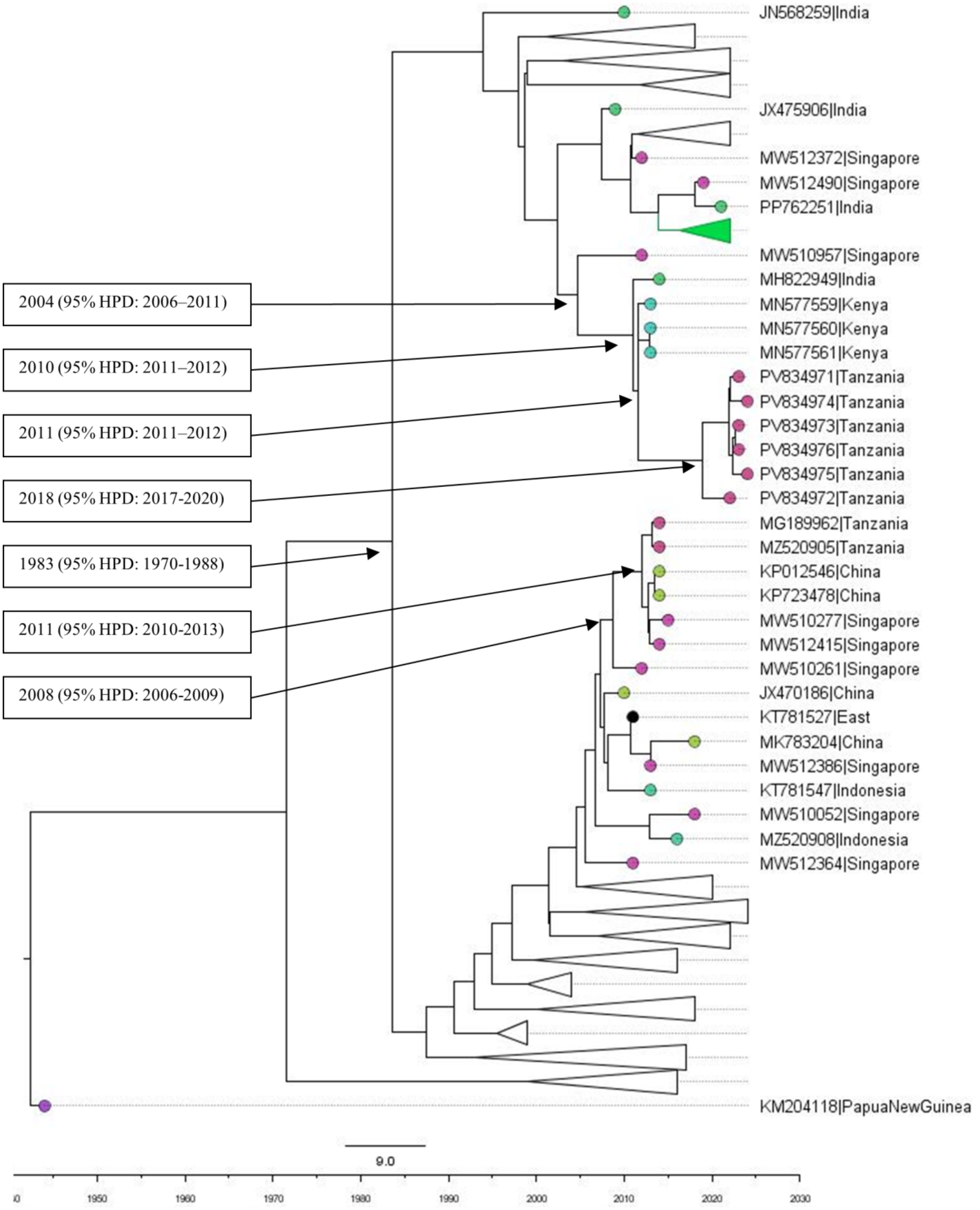

Figure S1. Bayesian phylogenetic analysis of the DENV-2 envelope (E) gene region was performed using a time-calibrated phylogeny, incorporating sampling tip dates to estimate divergence times. The nucleotide substitution model employed was HKY with gamma-distributed rate variation across four categories. A relaxed uncorrelated log-normal molecular clock was applied, with the mean clock rate fixed at 0.001 substitutions/site/year. The tree prior followed a coalescent Bayesian Skyline model to account for changes in effective population size over time. The Markov Chain

Monte Carlo (MCMC) analysis was run for 100 million iterations, sampling every 10,000 steps, with the first 10% discarded as burn-in. Convergence was assessed through effective sample size (ESS) values, with all key parameters exceeding 200. Posterior probabilities support key nodes, and divergence times are presented with 95% Highest Posterior Density (HPD) intervals. Phylogeny tips are coloured by country of origin to visualize the geographic distribution of sampled sequences.

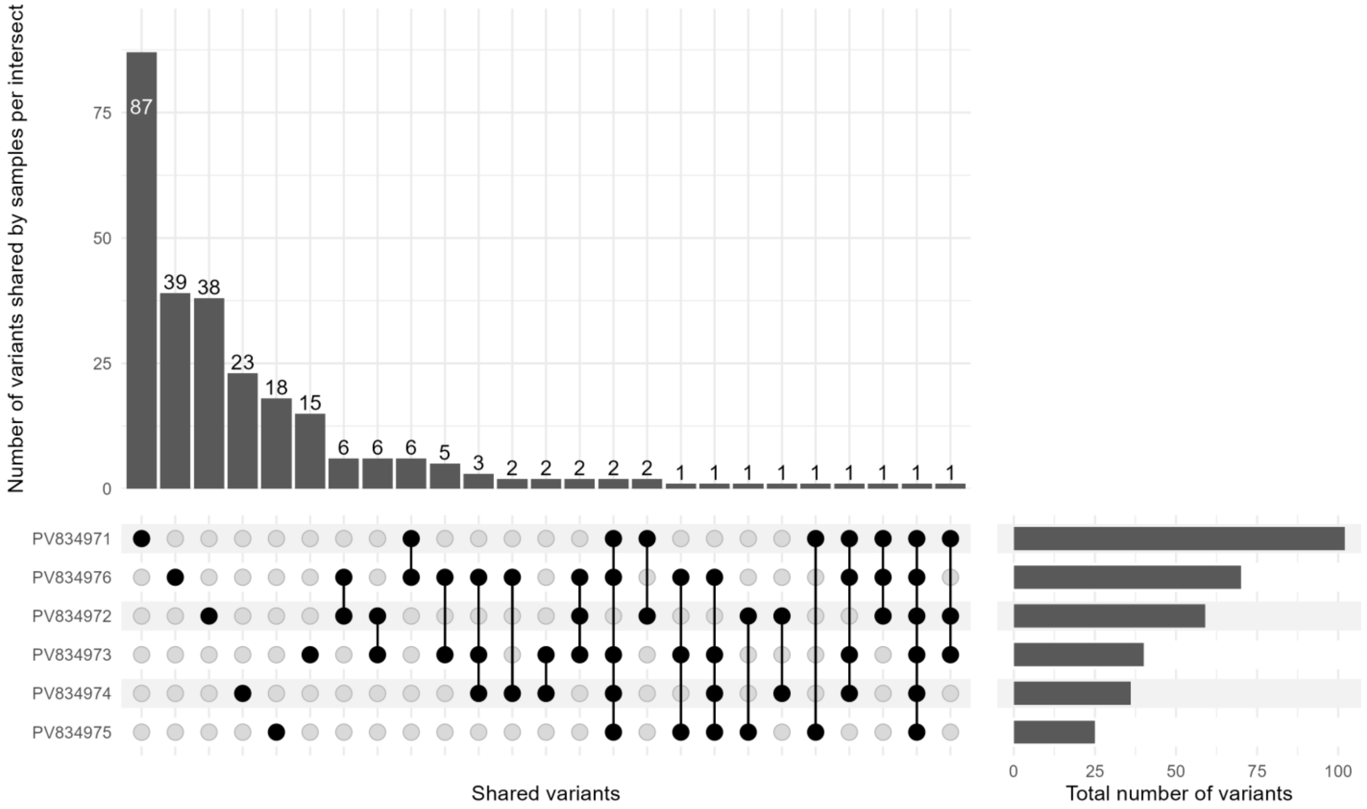

Figure S2. Distribution of shared variants across samples. Each bar in the intersection plot (top panel) represents the number of variants shared by the corresponding combination of samples indicated by the black dots in the lower matrix. Variants unique to individual samples are shown as single-sample bars, whereas multi-sample intersections highlight variants shared by two or more samples. The plot visualizes both unique and overlapping variant profiles, allowing comparison of variant sharing patterns across the cohort.

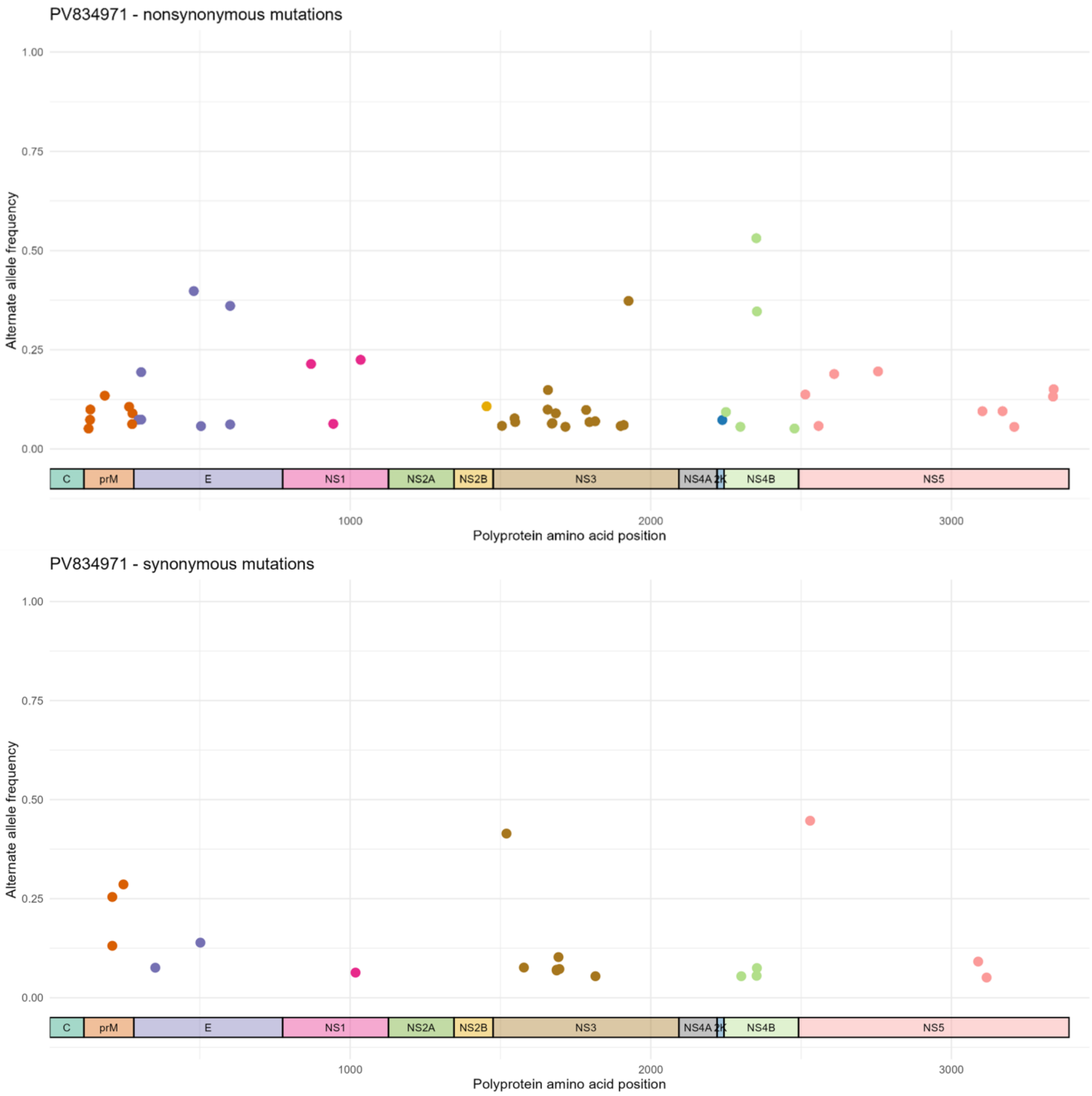

Figure S3. Variant frequencies of nonsynonymous and synonymous mutations PV834971. The upper panel displays the allele frequencies of nonsynonymous mutations whereas the lower the synonymous mutations. Coloured boxes below each graph indicate the genomic position of each mutation and its corresponding gene within the DENV-2 genome.

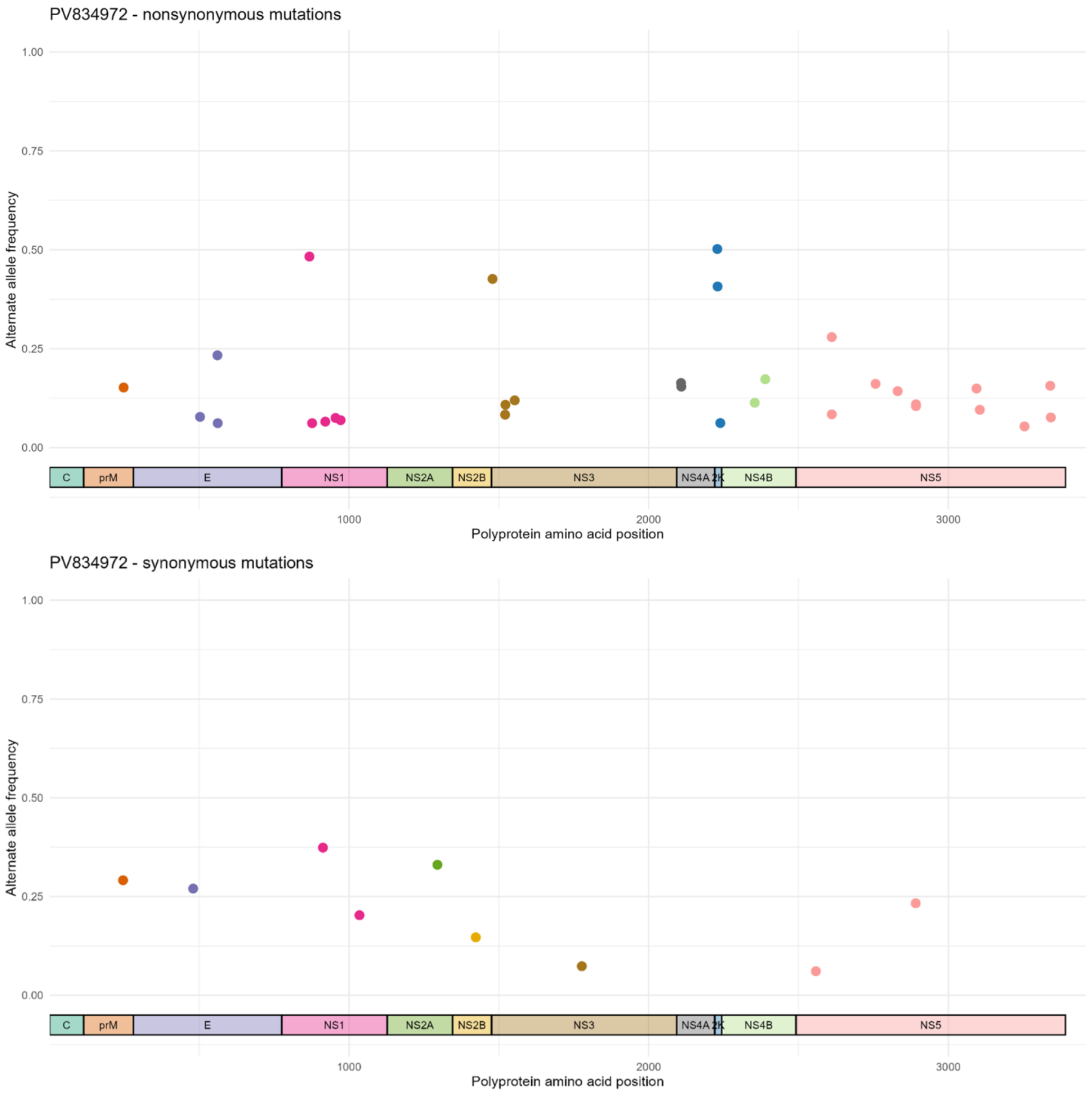

Figure S4. Variant frequencies of nonsynonymous and synonymous mutations PV834972. The upper panel displays the allele frequencies of nonsynonymous mutations whereas the lower the synonymous mutations. Coloured boxes below each graph indicate the genomic position of each mutation and its corresponding gene within the DENV-2 genome.

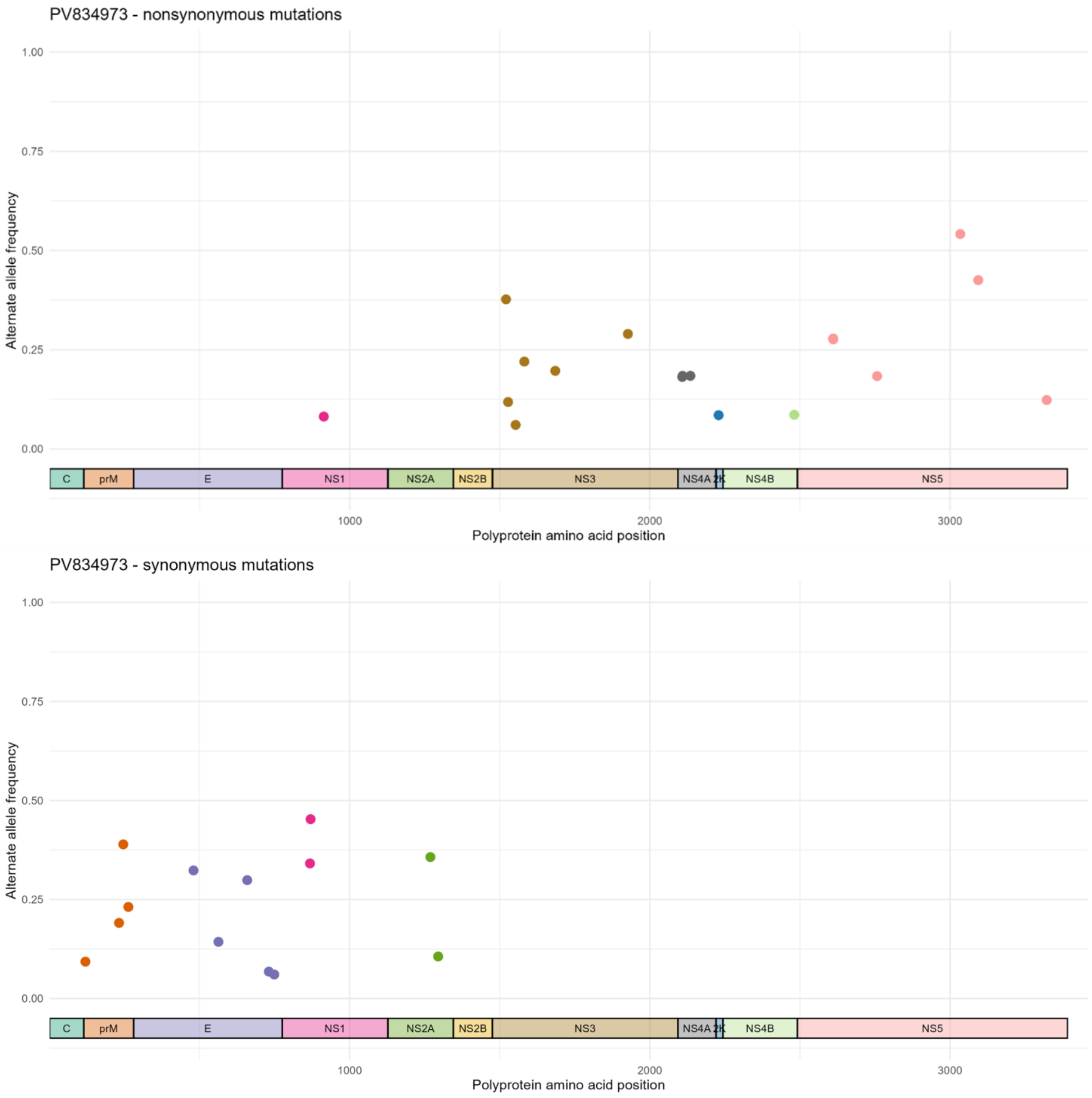

Figure S5. Variant frequencies of nonsynonymous and synonymous mutations PV834973. The upper panel displays the allele frequencies of nonsynonymous mutations whereas the lower the synonymous mutations. Coloured boxes below each graph indicate the genomic position of each mutation and its corresponding gene within the DENV-2 genome.

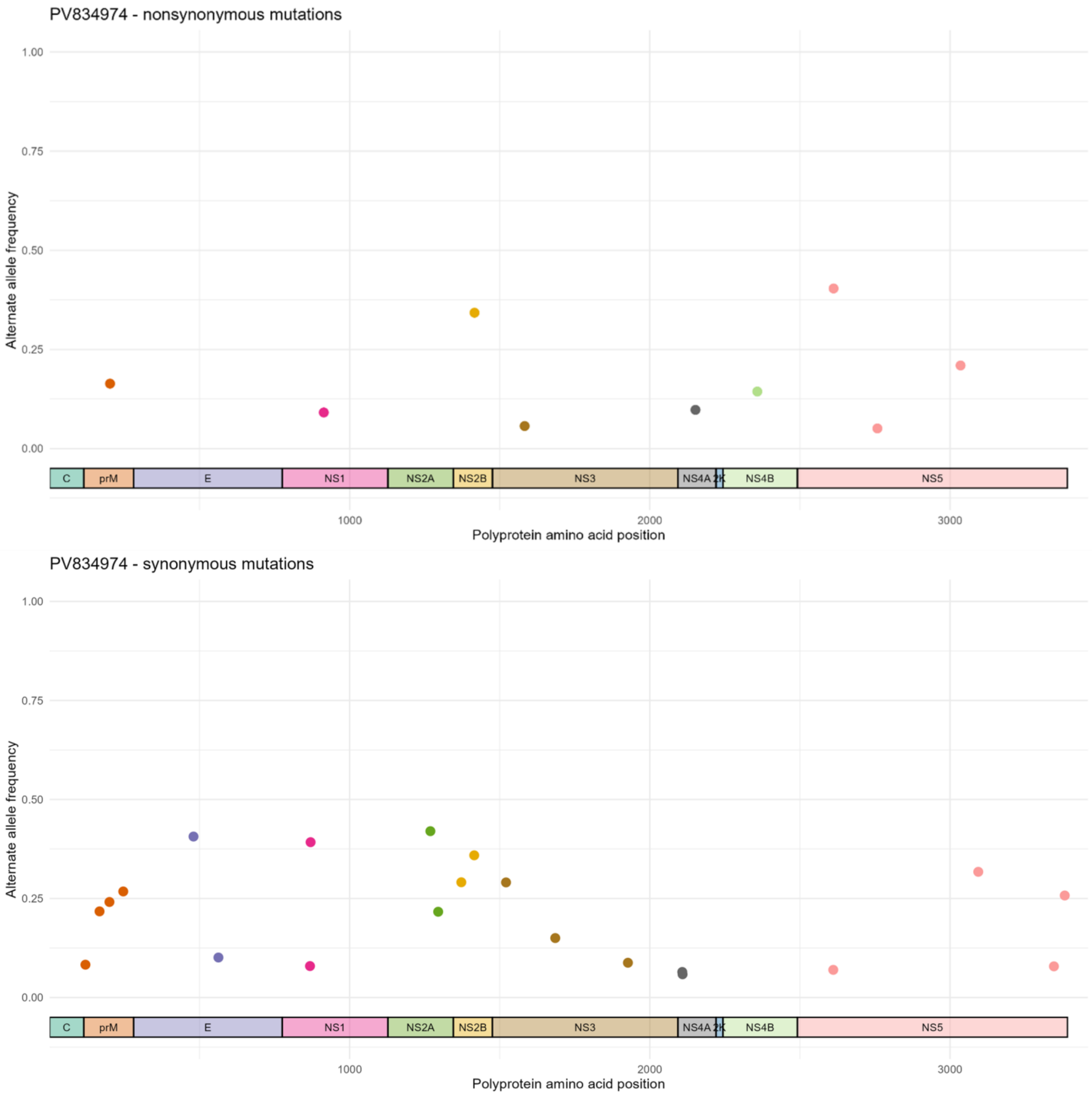

Figure S6. Variant frequencies of nonsynonymous and synonymous mutations PV834974. The upper panel displays the allele frequencies of nonsynonymous mutations whereas the lower the synonymous mutations. Coloured boxes below each graph indicate the genomic position of each mutation and its corresponding gene within the DENV-2 genome.

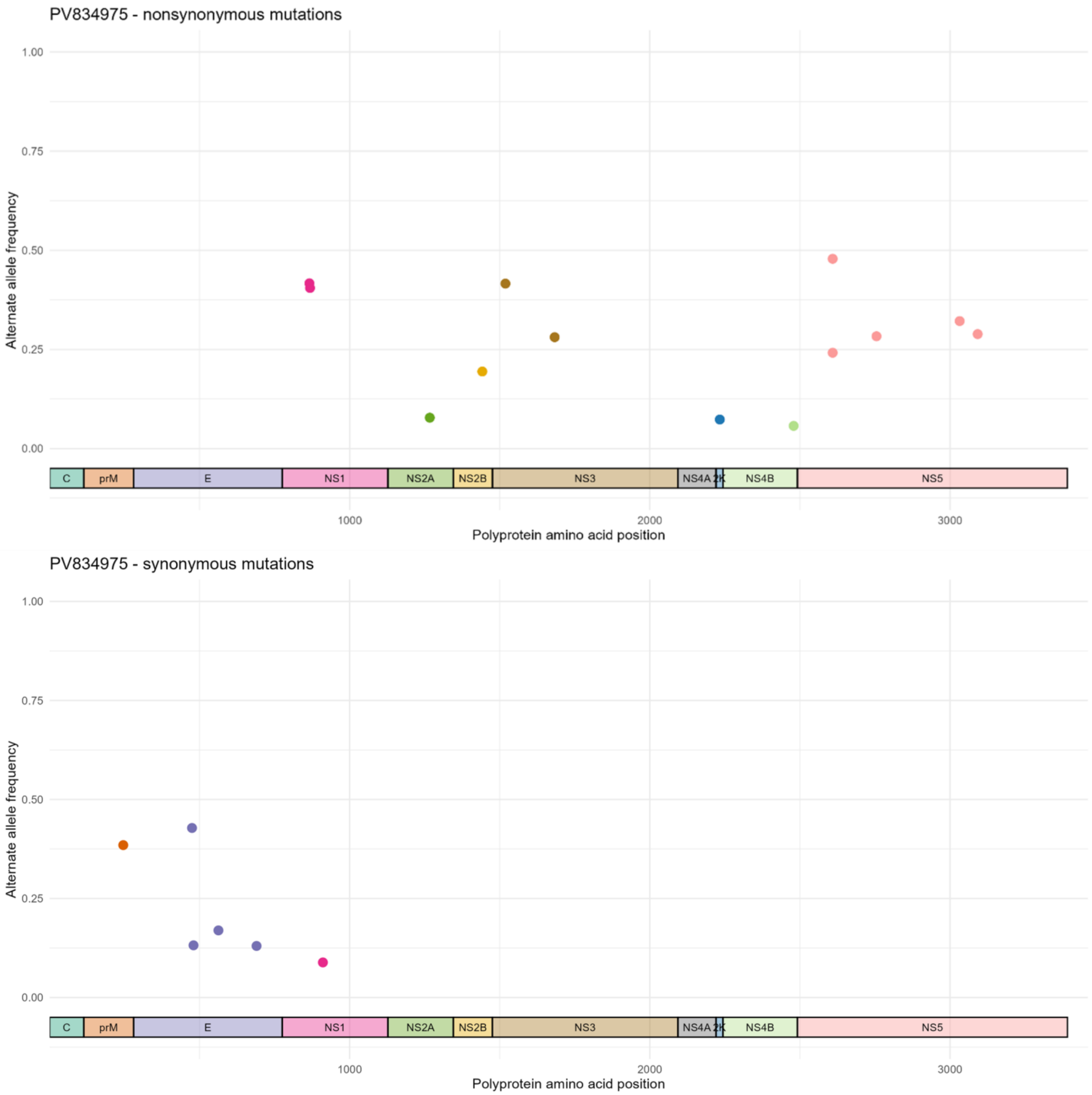

Figure S7. Variant frequencies of nonsynonymous and synonymous mutations PV834975. The upper panel displays the allele frequencies of nonsynonymous mutations whereas the lower the synonymous mutations. Coloured boxes below each graph indicate the genomic position of each mutation and its corresponding gene within the DENV-2 genome.

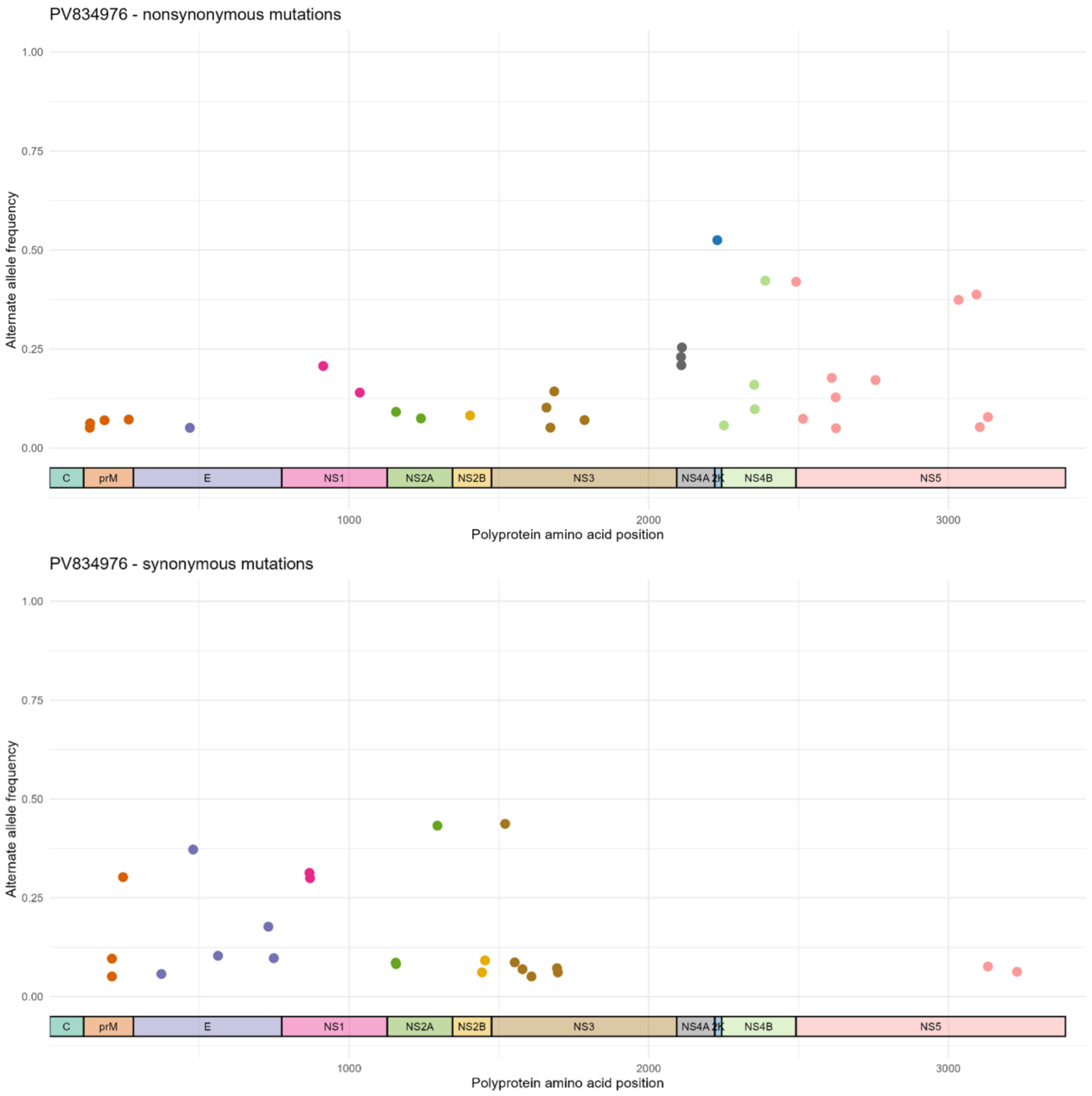

Figure S8. Variant frequencies of nonsynonymous and synonymous mutations PV834976. The upper panel displays the allele frequencies of nonsynonymous mutations whereas the lower the synonymous mutations. Coloured boxes below each graph indicate the genomic position of each mutation and its corresponding gene within the DENV-2 genome.

References

1. Katoh, K.; Standley, D.M. MAFFT multiple sequence alignment software version 7: improvements in performance and usability. *Mol. Biol. Evol.* **2013**, *30*, 772–780.
2. Rambaut, A. *FigTree v1.4.1. Institute of Evolutionary Biology*; University of Edinburgh: Edinburgh, UK, 2010.

Disclaimer/Publisher's Note: The statements, opinions and data contained in all publications are solely those of the individual author(s) and contributor(s) and not of MDPI and/or the editor(s). MDPI and/or the editor(s) disclaim responsibility for any injury to people or property resulting from any ideas, methods, instructions or products referred to in the content.
